# Plasma High Sensitivity Troponin T Levels in Adult Survivors of Childhood Leukaemias: Determinants and Associations with Cardiac Function

**DOI:** 10.1371/journal.pone.0077063

**Published:** 2013-10-21

**Authors:** Yiu-fai Cheung, Wei Yu, Daniel Ka-leung Cheuk, Frankie Wai-tsoi Cheng, Janet Yee-kwan Yang, Jeffrey Ping-wa Yau, Karin Ka-huen Ho, Chi-kong Li, Rever Chak-ho Li, Hui-leung Yuen, Alvin Siu-cheung Ling, Vivian Wing-yi Li, Wai-keung Wong, Kwong-cheong Tsang, Godfrey Chi-fung Chan

**Affiliations:** 1 Department of Paediatrics & Adolescent Medicine, Queen Mary Hospital, The University of Hong Kong, Hong Kong; 2 Department of Paediatrics, Prince of Wales Hospital, Hong Kong; 3 Department of Paediatrics and Adolescent Medicine, Tuen Mun Hospital, Hong Kong; 4 Department of Paediatrics, Queen Elizabeth Hospital, Hong Kong; 5 Department of Paediatrics and Adolescent Medicine, Princess Margaret Hospital, Hong Kong; 6 Department of Pathology and Clinical Biochemistry, Queen Mary Hospital, Hong Kong, China; Hospital Infantil Universitario Niño Jesús, Spain

## Abstract

**Background:**

We sought to quantify plasma high sensitivity cardiac troponin (hs-cTnT) levels, their determinants, and their associations with left ventricular (LV) myocardial deformation in adult survivors of childhood acute leukaemias.

**Methods and Results:**

One hundred adult survivors (57 males) of childhood acute leukaemias, aged 24.1±4.2 years, and 42 age-matched controls (26 males) were studied. Plasma cTnT was determined using a highly sensitive assay. Genotyping of NAD(P)H oxidase and multidrug resistance protein polymorphisms was performed. Left ventricular function was assessed by conventional, three-dimensional, and speckle tracking echocardiography. The medians (interquartile range) of hs-cTnT in male and female survivors were 4.9 (4.2 to 7.2) ng/L and 1.0 (1.0 to 3.5) ng/L, respectively. Nineteen survivors (13 males, 6 females) (19%) had elevated hs-cTnT (>95^th^ centile of controls). Compared to those without elevated hs-TnT levels, these subjects had received larger cumulative anthracycline dose and were more likely to have leukaemic relapse, stem cell transplant, and cardiac irradiation. Their LV systolic and early diastolic myocardial velocities, isovolumic acceleration, and systolic longitudinal strain rate were significantly lower. Survivors having CT/TT at *CYBA* rs4673 had higher hs-cTnT levels than those with CC genotype. Functionally, increased hs-cTnT levels were associated with worse LV longitudinal systolic strain and systolic and diastolic strain rates.

**Conclusions:**

Increased hs-cTnT levels occur in a significant proportion of adult survivors of childhood acute leukaemias and are associated with larger cumulative anthracycline dose received, history of leukaemic relapse, stem cell transplant, and cardiac irradiation, genetic variants in free radical metabolism, and worse LV myocardial deformation.

## Introduction

Survival of children with haematological malignancies has increased significantly with improved medical care and incorporation of anthracyclines into chemotherapy protocols [1]. With the population of adult survivors growing, recent long term follow-up series of childhood cancer survivors have further reaffirmed cardiovascular disorders as important causes of late morbidity and mortality [2], [3]. Identification of markers of early myocardial damage would facilitate stratification of patients into groups at varying risk of cardiac injury, which has implications on long term management. This is especially relevant as predisposition to anthracycline-induced cardiac damage, genetic and otherwise [4]–[6], varies among patients.

Damage to cardiomyocytes increases the circulating level of cardiac troponin T (cTnT), which provides the basis for using plasma or serum cTnT as a cardiac biomarker for diagnosing acute coronary syndrome [7], [8]. Intriguingly, circulating cTnT is detectable using standard assays in a small subset of general population [9], [10]. In these subjects who do not have overt cardiovascular disease, detectable cardiac troponins have been associated with increased risk of death and adverse cardiovascular risk profile and outcomes [9], [10]. Detectable circulating cTnT, albeit at a low level, may hence reflect subclinical myocardial injury [7]–[11]. Furthermore, these reported associations suggest that cTnT may be useful for detection of subclinical cardiovascular disease in individuals at increased cardiovascular risk, although the relative low sensitivity of standard assays has limited the usefulness of this potential application. New generation of highly sensitive assay has enabled the measurement of very low circulating levels of cTnT at approximately 10 fold lower than those detectable with conventional assays [12]–[14]. In the general population, cTnT level as quantified using the highly sensitive assay (hs-cTnT) has been associated with cardiovascular risk factors and structural alteration of the left ventricle, and shown to predict cardiovascular risk and subsequent risk for all-cause mortality [12], [13]. In patients with chronic heart failure, changes in hs-cTnT concentrations over time have also been shown to predict future cardiovascular events [15].

In the present study, we sought to quantify plasma hs-cTnT levels and determine factors associated with elevated hs-cTnT levels in adult survivors of childhood leukaemias. We further explored associations between plasma hs-cTnT levels and indices of left ventricular (LV) function and myocardial deformation in our survivors who did not have overt cardiac failure.

## Methods

### Ethics Statement

All subjects gave written informed consent to participate in this study approved by the Institutional Review Board of The University of Hong Kong/Hospital Authority West Cluster, Hong Kong.

### Subjects

One hundred anthracycline-treated adult survivors of childhood leukaemias were recruited territory-wide from five public hospitals in Hong Kong. These five hospitals treated nearly all childhood cancers in Hong Kong. Inclusion criteria included i) survivors aged 18 to 35 years old, ii) history of acute leukaemia diagnosed before 18 years old, and iii) completion of cancer directed therapy for at least 5 years. Exclusion criteria included i) history of congenital heart disease, ii) syndromal disorders associated with congenital heart disease, and iii) hypothyroidism not on replacement therapy. Data collection from the case notes included the following: diagnosis, age at diagnosis, date of completion of chemotherapy, cumulative anthracycline dose, history of relapse, history of cardiac irradiation, whether stem cell transplant has been performed, major organ dysfunction, and current cardiac medications. Forty-two adult healthy subjects, including volunteer staff members, their healthy friends and relatives, and healthy siblings of survivors, were recruited as controls.

The body weight and height were measured and the body mass index and body surface area were calculated accordingly. All subjects rest for at least 15 minutes before echocardiographic assessments, followed immediately by blood taking for assay of hs-cTnT and genotyping as described below.

### Echocardiographic Assessment

All echocardiographic assessments were performed using the Vivid 7 ultrasound machine (GE Medical System, Horten, Norway). The digital acquisitions were analyzed offline using the EchoPAC software (GE Medical System, Horten, Norway). The average value of the echocardiographic indices from three cardiac cycles was calculated. All of the echocardiographic acquisitions and analyses were performed by one single investigator (Wei Yu).

Parasternal short-axis view at mid-LV level was performed to derive the following M-mode measurements: LV end-systolic and end-diastolic dimensions, and thickness of interventricular septum and posterior LV wall. The fractional shortening and the mass of the left ventricle were calculated according to standard formulae. Transmitral pulsed-wave Doppler examination was performed to obtain the early (E) and late (A) inflow velocities and E deceleration time. Tissue Doppler imaging was done with the sample volume positioned at basal LV free wall-mitral annular junction to obtain the peak systolic (s), and early (e) and late (a) diastolic myocardial tissue velocities, e/a ratio, and LV free wall myocardial acceleration during isovolumic contraction (IVA), the latter being a relative load-independent index of LV contractile function [16].

Speckle tracking echocardiography was used to assess global LV longitudinal, radial, and circumferential strain and strain rate (SR) as reported previously [17]. Briefly, the four-chamber apical plane was acquired for assessment of LV longitudinal strain and strain rate, while the short-axis plane at the papillary muscle level was acquired for assessment of LV radial and circumferential strain and strain rate. Additionally, three-dimensional echocardiography was performed using the matrix ultrasound probe for derivation of LV systolic and diastolic volumes and ejection fraction as previously described [17].

### Blood Investigations

Venous blood was collected using EDTA tubes for DNA extraction and plasma samples were stored at −80°C until assay. Plasma cTnT was measured using a highly sensitive assay (Elecsys-2010 Troponin T hs, Roche Diagnostics), which has a lower detection limit of 3 ng/L. Genotyping for polymorphism of NAD(P)H oxidase (*CYBA* rs4673, *RAC2* rs13058338, *NCF4* rs1883112) and multidrug resistance protein (*MRP1* rs45511401, *MRP2* rs17222723, *MRP2* rs8187710), reported previously to be associated with acute or chronic anthracycline-induced cardiotoxicity [5] was performed. All allelic discrimination was performed by ABI 7900HT Real-Time PCR system using pre-developed TaqMan SNP genotyping assay (Applied Biosystems, Foster City, CA USA), except for *MRP1* rs45511401. *MRP1* rs45511401 genotyping was performed by PCR-direct sequencing. Briefly, PCR fragments were amplified and followed by sequencing with ABI 3730×l DNA Analyzer. Genotypes were determined by homology analysis using BLAST (http://blast.ncbi.nlm.nih.gov/Blast.cgi).

### Statistical Analysis

Data are presented as mean±SD unless otherwise stated. Left ventricular dimensions and volumes were indexed by body surface area. Demographic variables and echocardiographic parameters of survivors and controls were compared using Student’s *t* test and Fisher’s exact test where appropriate. Comparison of plasma hs-cTnT levels between survivors and controls, which had a non-parametric distribution, was performed using Mann-Whitney U test. The 95^th^ percentiles of hs-cTnT in male and female healthy controls were determined by non-parametric estimation. Male and female survivors were then stratified into two groups based on the respective cutoffs: group I with increased hs-cTnT levels >95^th^ centile and group II with hs-cTnT levels ≤95^th^ centile. Differences in variables between the two survivor subgroups were compared using Student’s *t* test and Fisher’s exact test where appropriate. A value of 1 ng/L was assigned for hs-cTnT levels below the limit of detection (<3 ng/L), which would give a value of 0 after logarithmic transformation. Logarithmic transformation of hs-cTnT levels was performed to normalize the distribution of hs-cTnT levels for correlation analyses. Within the survivor group, correlations between natural log-transformed hs-cTnT levels and indices of myocardial deformation and cumulative anthracycline dose were determined by Pearson correlation analysis. Stepwise multiple linear regression analysis was performed to identify significant determinants of natural log-transformed hs-cTnT level in survivors. Genotypic and allelic frequencies were compared between survivors and controls using Fisher’s exact test. A p value <0.05 was considered statistically significant. All statistical analyses were performed using IBM SPSS Statistics Version 19 (U.S.A).

## Results

### Subjects

The 100 survivors (57 males), aged 24.1±4.2 years, were studied at 15.3±5.8 years after completion of cancer treatment. Their age at diagnosis was 8.0±5.0 years. All had received anthracycline as part of the chemotherapeutic regimen for the treatment of acute lymphoblastic (n = 88) or myeloid (n = 12) leukaemia. The mean cumulative anthracycline dose was 218±98 mg/m^2^ (median 220, range 10 to 541 mg/m^2^). Of the 100 survivors, 23 had leukaemic relapse and 15 had undergone stem cell transplant. Thirteen survivors, 12 of whom had stem cell transplant and 1 had central nervous system leukaemia, had received irradiation involving the heart. All of the survivors were free from cardiac symptoms. One survivor received amlodipine for systemic hypertension. Associated medical problems included hypogonadism in 11, growth hormone deficiency in 1, hypoadrenalism in 1, hypothyroidism with replacement therapy in 1, and development of parotid acinic cell carcinoma in 1. The 42 controls (26 males) were aged 25.4±4.1 years. While the age (p = 0.10) and genders (p = 0.82) were similar between survivors and controls, the survivors had significantly lower body weight (p = 0.046), height (p<0.001), and body surface area (p = 0.006) (Table 1).

**Table 1:** Comparisons of demographic and echocardiographic findings between survivors and control subjects.

	Patients	Controls	p
	(n = 100)	(n = 42)	
*Demographics*			
Age (years)	24.1±4.2	25.4±4.1	0.10
Sex (M:F)	57∶43	26∶16	0.82
Weight (kg)	59.7±12.1	64.1±11.3	0.046*
Height (cm)	164.5±8.8	170.1±7.6	<0.001*
Body surface area (m^2^)	1.6±0.2	1.7±0.2	0.006*
*M-mode echocardiographic indices*			
Indexed LVEDd (mm)	29.7±3.6	30.4±3.0	0.31
Indexed LVESd (mm)	20.5±2.6	20.0±2.2	0.30
FS (%)	31.1±3.8	33.9±3.6	<0.001*
Indexed LV mass (g/m^2^)	66.9±16.9	69.3±18.6	0.45
*3D echocardiographic indices*			
LV end-systolic volume (ml/m^2^)	32.7±9.2	27.9±6.8	0.003*
LV end- diastolic volume (ml/m^2^)	70.1±16.7	63.7±13.2	0.032*
LV EF (%)	53.2±7.2	56.0±7.4	0.042*
*Doppler indices*			
E (cm/s)	88.0±17.1	84.3±15.3	0.24
A (cm/s)	48.7±12.6	45.5±11.4	0.18
E/A ratio	1.9±0.6	2.0±0.5	0.82
E deceleration time (ms)	172.7±34.6	163.4±32.3	0.15
s (cm/s)	8.2±1.9	8.4±1.9	0.66
e (cm/s)	−12.0±2.0	−11.8±2.4	0.62
a (cm/s)	−5.1±1.8	−4.8±1.6	0.18
e/a	2.7±1.1	2.7±1.2	0.97
IVA (m/s^2^)	1.19±0.49	1.27±0.63	0.42
*Deformation indices*			
Longitudinal strain (%)	−16.0±3.1	17.1±3.2	0.049*
Longitudinal SRs(/s)	−0.93±0.18	−0.94±0.20	0.68
Longitudinal SRd(/s)	1.28±0.32	1.34±0.32	0.30
Circumferential strain (%)	−14.3±3.5	−16.6±4.7	0.001*
Circumferential SRs(/s)	−0.94±0.24	−1.06±0.33	0.027*
Circumferential SRd(/s)	1.12±0.39	1.27±0.45	0.049*
Radial strain (%)	32.9±10.9	42.3±12.5	<0.001*
Radial SRs(/s)	1.90±0.49	2.14±0.57	0.003*
Radial SRd(/s)	−1.88±0.67	−1.99±0.67	0.36

Abbreviations: A, peak mitral inflow velocity at late diastole; a, mitral annular late diastolic myocardial tissue velocity; E, peak mitral inflow velocity at early diastole; e, mitral annual early diastolic myocardial tissue velocity; EF, ejection fraction; FS, fractional shortening; IVA, isovolumic acceleration; LV, left ventricular; LVEDd, left ventricular end diastolic dimension; LVESd, left ventricular end systolic dimension; SR_d_, early diastolic strain rate; SR_s_, systolic strain rate.

* statistically significant.

### Echocardiographic Parameters

Table 1 summarizes the echocardiographic findings in survivors and controls. Compared with controls, survivors had slightly lower, albeit statistically significant, LV shortening fraction (p<0.001) and ejection fraction (p = 0.042), but similar Doppler indices of LV systolic and diastolic function (all p>0.05). For speckle tracking parameters, survivors had significantly lower global LV longitudinal systolic strain (p = 0.049), circumferential systolic strain (p = 0.001) and systolic (p = 0.027) and diastolic (p = 0.049) strain rates, and radial systolic strain (p<0.001) and systolic strain rate (p = 0.003) than controls.

### Prevalence and Levels of Detectable hs-cTnT

**Figure 1:**
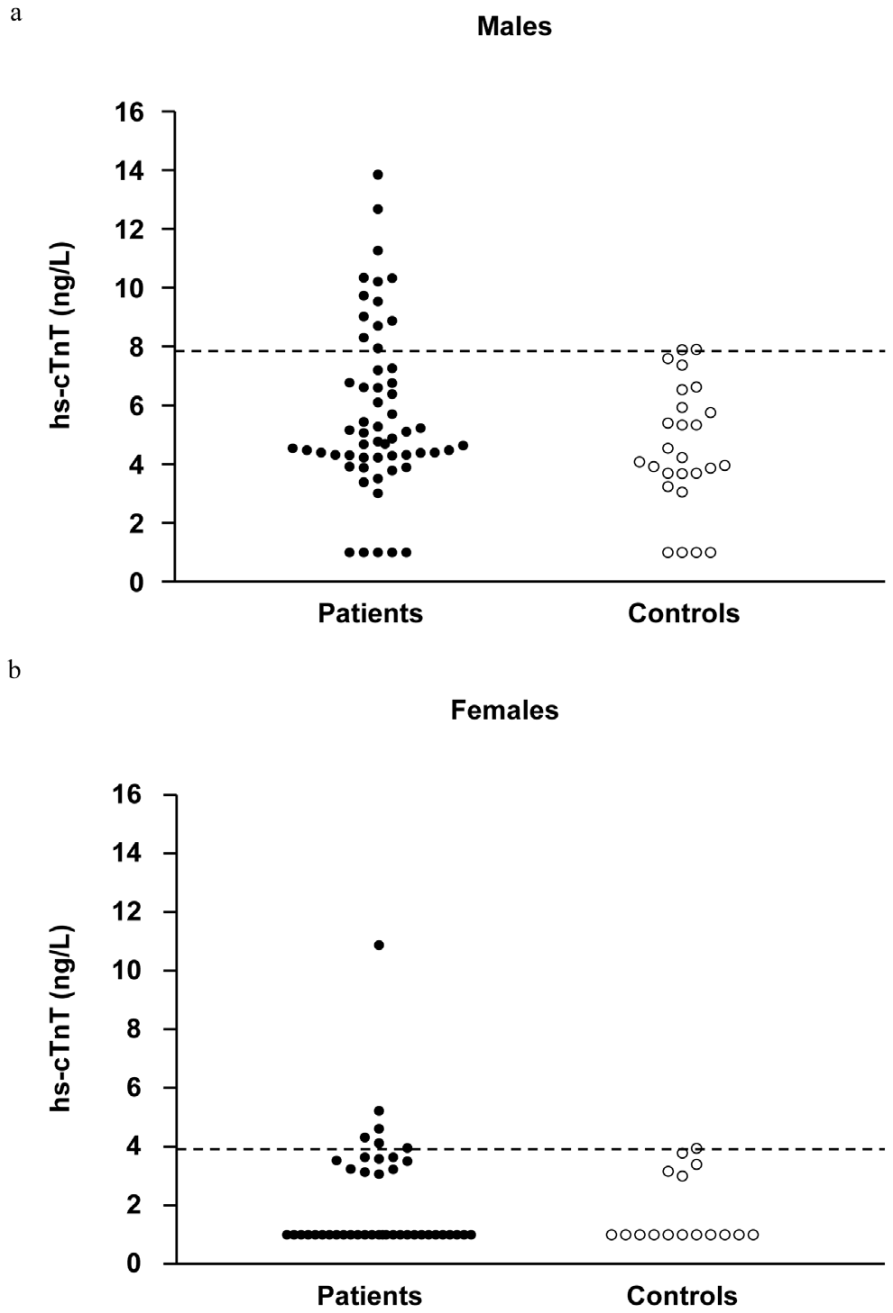
Levels of high sensitivity cardiac troponin T (hs-cTnT) in survivors and controls by genders. Distributions of hs-cTnT in (a) male and (b) female survivors and controls are shown using scatter plots. Dashed lines represent 95^th^ percentile in controls.

For males, the prevalence of detectable plasma hs-cTnT in survivors was 91.2% (95% CI, 81.1 to 96.2%) versus 84.6% (95% CI, 66.5 to 93.9%) in controls (p = 0.45). For females, the prevalence of detectable plasma hs-cTnT was 34.9% (95% CI, 22.4 to 49.8%) in survivors versus 31.3% (95% confidence interval, 14.2 to 55.6%) in controls (p = 1.0). The distribution of plasma hs-cTnT levels in survivors and controls is shown in figure 1. The 95^th^ percentile of plasma hs-cTnT level in healthy controls as defined by nonparametric estimate was 7.91 ng/L in males and 3.95 ng/L in females. Based on these cutoff values, a total of 19 (13 males and 6 females) survivors were found to have elevated plasma hs-cTnT levels, accounting for an overall prevalence of 19.0% (95% CI, 12.5 to 27.8%).

### Plasma hs-cTnT Levels and Clinical Variables

Comparisons of clinical variables by univariate analyses between survivors with elevated hs-cTnT levels (group I) and those without (group II) are summarized in Table 2. The two groups shared similar demographic characteristics and types of leukaemias (all p>0.05). However, the cumulative anthracycline dose received by group I survivors was significantly higher (p<0.001). More survivors in group I than in group II had leukaemic relapse (p = 0.037), undergone stem cell transplant (p = 0.036), and received irradiation involving the heart (p = 0.016).

**Table 2:** Comparisons of demographic factors, clinical variables, and genotypic frequencies between survivors with (group I) and without (group II) elevated hs-cTnT levels.

	Group I	Group II	p
	(n = 19)	(n = 81)	
*Demographics*			
Age (years)	24.7±3.7	24.0±4.3	0.52
Sex (M:F)	13∶6	44∶37	0.31
Weight (kg)	59.8±11.6	59.6±12.3	0.97
Height (cm)	166.4±7.7	164.0±9.0	0.28
Body surface area (m^2^)	1.7±0.2	1.6±0.2	0.66
*Clinical variables*			
ALL:AML	15∶4	73∶8	0.23
Cumulative doxorubicindose (mg/m^2^)	288±126	201±83	<0.001*
Irradiation involvingthe heart (Y:N)	6∶13	7∶74	0.016*
Relapse (Y:N)	8∶11	15∶66	0.037*
Stem cell transplant (Y:N)	6∶13	9∶72	0.036*
*Genotypes*			
* CYBA* (CC:CT/TT)	14∶5	72∶9	0.13
* RAC2* (TT:TA/AA)	15∶4	62∶19	1.0
* NCF4* (GG/GA:AA)	10.9	47∶34	0.80

Abbreviations: AML, acute myeloid leukaemia; ALL, acute lymphoblastic leukaemia.

* statistically significant.

The plasma hs-cTnT levels were significantly greater in survivors who had irradiation involving the heart (median 5.23 ng/L vs 3.90 ng/L, p = 0.046), and also tended to be higher in those with stem cell transplant (median 4.89 ng/L vs 3.88 ng/L, p = 0.06) and leukaemic relapse (median 4.55 ng/L vs 3.65 ng/L, p = 0.059) than those without. Natural log-transformed plasma hs-cTnT levels also correlated significantly with cumulative anthracycline dose (r = 0.24, p = 0.011). Multivariate analysis revealed that the significant correlates of plasma hs-cTnT levels were gender (β = −0.63, p<0.001; males having higher hs-cTnT levels) and cumulative anthracycline dose (β = 0.24, p = 0.002) after adjustment for age at diagnosis, age at study, body mass index, and history of leukaemic relapse, irradiation involving the heart, and stem cell transplant.

### Genotypes and hs-cTnT Levels

The genotypic and allelic frequencies of NAD(P)H oxidase and multidrug resistance protein genes in survivors and controls, excluding the four healthy siblings of survivors, are shown in table 3. In our ethnic Chinese subjects, none were carrying the variant alleles of the multidrug resistance proteins *MRP1* and *MRP2*. Genotypic and allelic frequencies of *CYBA* and *RAC2* were similar between survivors and controls. The *NCF4* GG/AG genotype was more prevalent than AA genotype in survivors than controls (p = 0.038).

**Table 3:** Genotypic and allelic frequencies of NAD(P)H oxidase and multidrug resistance protein genes in survivors and control subjects, and genotypic frequencies reported in Chinese population from National Center for Biotechnology Information (NCBI) database.

Genotypes	Patients	Controls	OR(95% CI)	p	NCBI
	(n = 100)	(n = 38**)			database
*CYBA* *(rs4673)*			1.64(0.63–4.29)a	0.31	
CC	86 (86.0%)	30 (78.9%)			86%
CT	13 (13.0%)	8 (21.1%)			14%
TT	1 (1.0%)	0 (0%)			0%
*RAC2* *(rs13058338)*			1.36(0.59–3.17)b	0.51	
TT	77 (77.0%)	27 (71.1%)			86.7%
AT	21 (21.0%)	11 (28.9%)			13.3%
AA	2 (2.0%)	0 (0%)			0%
*NCF-4* *(rs1883112)*			2.27(1.05–4.90)c	0.038*	
GG	15 (15.0%)	2 (5.2%)			9.3%
AG	42 (42.0%)	12 (31.6%)			34.9%
AA	43 (43.0%)	24 (63.2%)			55.6%
*MRP1* *(rs45511401)*			−	−	
GG	100 (100%)	38 (100%)			
GT	0 (0%)	0 (0%)			
TT	0 (0%)	0 (0%)			
*MRP2* *(rs17222723)*			−	−	
TT	100 (100%)	38 (100%)			
TA	0 (0%)	0 (0%)			
AA	0 (0%)	0 (0%)			
*MRP2* *(rs8187710)*			−	−	
GG	100 (100%)	38 (100%)			100%
GA	0 (0%)	0 (0%)			0%
AA	0 (0%)	0 (0%)			0%
**Alleles**	**Patients**	**Controls**	**OR** **(95% CI)**	**p**	
	**(n = 100)**	**(n = 38** ** **)**			
*CYBA* *(rs4673)*			1.45(0.59–358)	0.47	
C	185	68			
T	15	8			
*RAC2* *(rs13058338)*			1.19(0.55–2.54)	0.69	
T	175	65			
A	25	11			
*NCF-4* *(rs1883112)*			2.11(1.13–3.93)	0.02*	
G	72	16			
A	128	60			
*MRP1* *(rs45511401)*			–	–	
G	200	76			
T	0	0			
*MRP2* *(rs17222723)*			–	–	
T	200	76			
A	0	0			
*MRP2* *(rs8187710)*			–	–	
G	200	76			
A	0	0			

* statistically significant.

** 4 controls who were siblings of patients were excluded.

a CC vs CT/TT.

b TT vs AT/AA.

c GG/AG vs AA.

**Figure 2:**
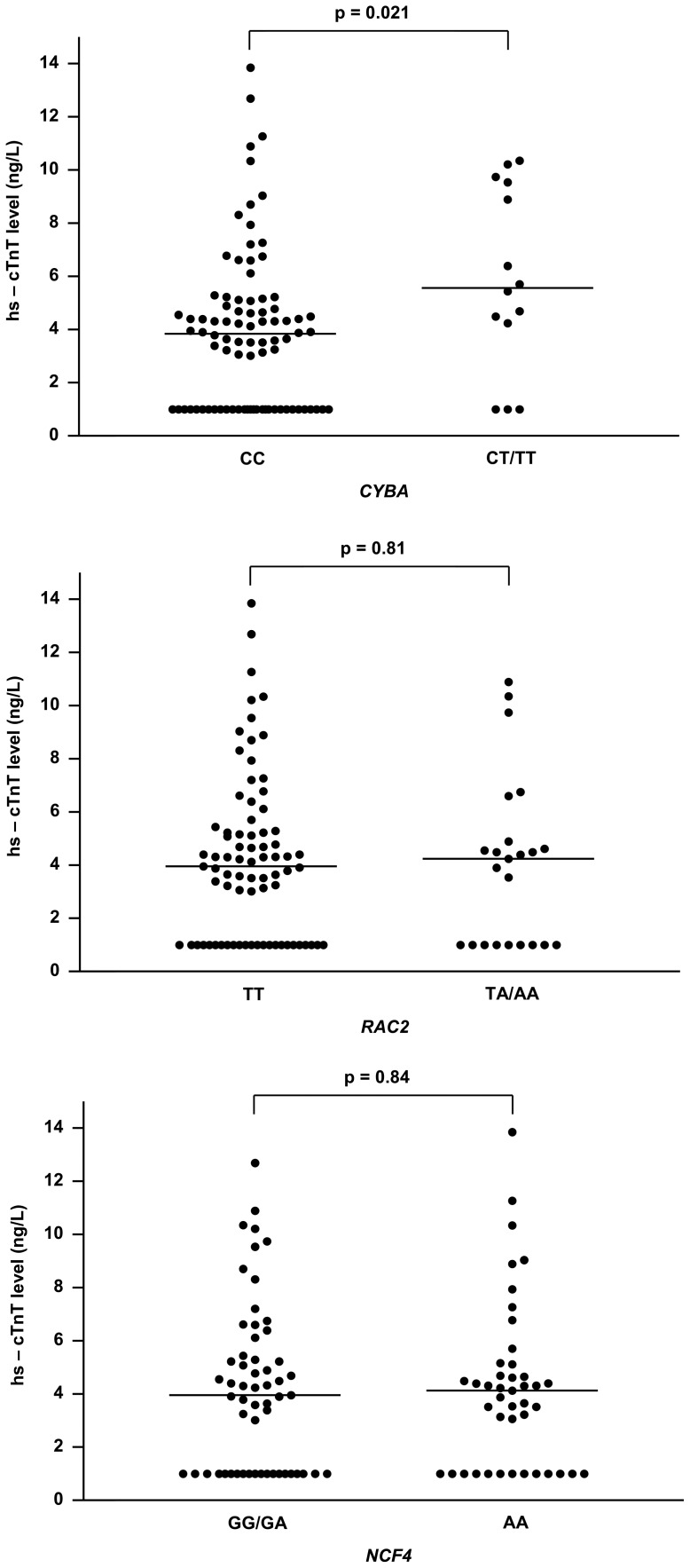
Levels of high sensitivity cardiac troponin T (hs-cTnT) in survivors by NAD(P)H oxidase and multidrug resistance protein polymorphisms. Scatter plots showing distributions of hs-cTnT levels in relation to (a) *CYBA*, (b) *RAC2*, and (c) *NCF4* polymorphisms. Solid lines represent median hs-cTnT levels.

The genotypic frequencies of *CYBA*, *RAC2*, and *NCF4* were similar between group I and group II survivors (Table 2). Stratified by genetic variants, plasma hs-cTnT levels were significantly greater in subjects with CT/TT than those with CC genotype at *CYBA* rs4673 (Fig. 2). Variants in *RAC2* and *NCF4* were not associated with increased hs-cTnT levels.

### Plasma hs-cTnT Levels and Cardiac Parameters

Comparisons of echocardiographic parameters between group I and group II survivors are summarized in Table 4. Compared with group II survivors, group I survivors had significantly greater indexed LV mass (p = 0.008), lower systolic (p = 0.041) and early diastolic (p = 0.045) mitral annular velocities, reduced LV IVA (p = 0.007) and lower longitudinal systolic strain rate (p = 0.049), but similar LV ejection fraction (p = 0.19).

**Table 4:** Comparisons of echocardiographic findings between survivors with (group I) and without (group II) elevated hs-cTnT levels.

	Group I	Group II	p
	(n = 19)	(n = 81)	
*M-mode echocardiographic indices*			
Indexed LVEDd (mm)	30.4±3.8	29.5±3.6	0.33
Indexed LVESd (mm)	20.6±3.0	20.5±2.5	0.83
FS (%)	32.0±3.1	30.9±4.0	0.24
Indexed LV mass (g/m^2^)	76.0±23.4	64.6±14.3	0.008*
*3D echocardiographic induces*			
LV end-systolic volume(ml/m^2^)	34.5±11.4	32.3±8.6	0.37
LV end- diastolic volume(ml/m^2^)	71.0±18.1	69.9±16.4	0.81
LV EF (%)	51.3±6.6	53.7±7.3	0.19
*Doppler indices*			
E (cm/s)	86.4±15.4	88.4±17.5	0.65
A (cm/s)	51.2±12.1	48.0±12.7	0.32
E/A ratio	1.8±0.6	2.0±0.6	0.30
E deceleration time (ms)	171.6±38.4	172.9±33.9	0.88
s (cm/s)	7.4±1.8	8.4±1.8	0.041*
e (cm/s)	−11.2±2.5	−12.2±1.8	0.045*
a (cm/s)	−4.4±1.6	−5.3±1.8	0.052
e/a	2.8±1.0	2.7±1.2	0.57
IVA (m/s^2^)	0.92±0.37	1.26±0.50	0.007*
*Deformation indices*			
Longitudinal strain (%)	−15.2±2.5	−16.2±3.2	0.19
Longitudinal SRs (/s)	−0.86±0.14	−0.95±0.18	0.049*
Longitudinal SRd (/s)	1.24±0.33	1.29±0.32	0.59
Circumferential strain (%)	−14.2±3.7	−14.3±3.5	0.90
Circumferential SRs (/s)	−0.93±0.23	−0.95±0.24	0.74
Circumferential SRd (/s)	1.08±0.27	1.13±0.41	0.62
Radial strain (%)	32.2±7.2	33.0±11.7	0.79
Radial SRs (/s)	1.74±0.39	1.88±0.51	0.26
Radial SRd (/s)	−1.96±0.90	−1.86±0.61	0.53

Abbreviations: A, peak mitral inflow velocity at late diastole; a, mitral annular late diastolic myocardial tissue velocity; E, peak mitral inflow velocity at early diastole; e, mitral annual early diastolic myocardial tissue velocity; EF, ejection fraction; FS, fractional shortening; IVA, isovolumic acceleration; LV, left ventricular; LVEDd, left ventricular end diastolic dimension; LVESd, left ventricular end systolic dimension; SR_d_, early diastolic strain rate; SR_s_, systolic strain rate.

* statistically significant.

**Figure 3:**
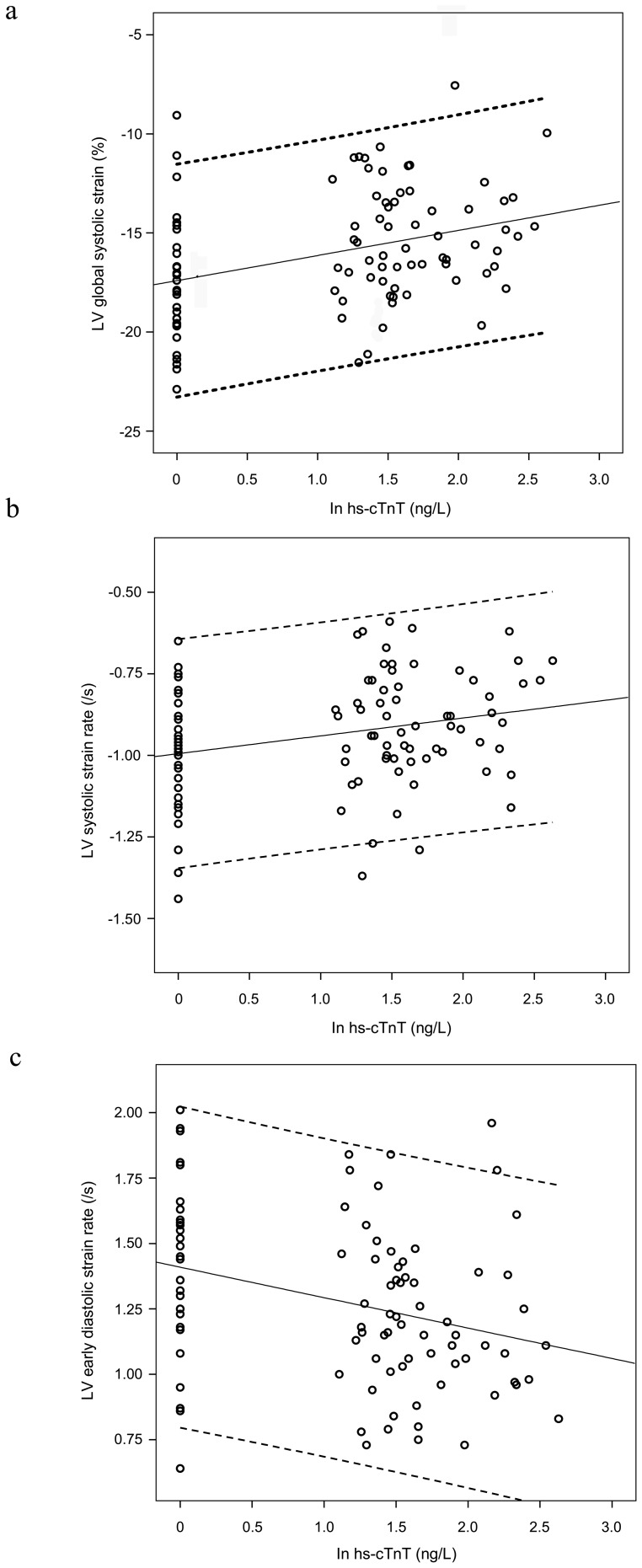
Relationships between high sensitivity cardiac troponin T (hs-cTnT) and indices of left ventricular (LV) deformation. Correlations between hs-cTnT and LV (a) global longitudinal systolic strain, (b) systolic strain rate, and (c) diastolic strain rate are shown. Solid lines represent the best-fit linear regression and dashed lines represent the 95% confidence interval for each parameter of the regression line.

Treating hs-cTnT level as a continuous variable, significant correlations were found between natural log-transformed hs-cTnT levels and LV mass indexed by body surface area (r = 0.28, p = 0.006), IVA (r = −0.24, p = 0.016), and global longitudinal systolic strain (r = 0.35, p = 0.001), systolic strain rate (r = 0.26, p = 0.011), and diastolic strain rate (r = −0.31, p = 0.002) (Figure 3), but not with LV shortening fraction (p = 0.76) and ejection fraction (p = 0.27).

## Discussion

The present study provides the first evidence of increased circulating hs-cTnT levels in a subgroup of adult survivors of childhood leukaemia, with a prevalence of 19% in our surviving cohort studied at a mean of about 15 years after completion of chemotherapy. Cumulative anthracycline dose received and history of leukaemic relapse, stem cell transplant, and irradiation involving the heart are associated with elevated hs-cTnT levels in univariate and multivariate analyses. Polymorphism of the *CYBA* rs4673 is found also to be associated with higher hs-cTnT levels. Functional implications of increased hs-cTnT level are reflected by its associations with worse LV systolic and diastolic myocardial deformation.

Exploration of the changes in circulating cardiac biomarkers in childhood cancer patients has been limited [18], [19]. Elevated cTnT levels, as assessed using standard assays, after initial doxorubicin therapy for acute lymphoblastic leukaemia in children has been shown to associate with LV dilation and wall thinning 9 months later [18] and reduced LV mass 4 years later [19]. In the first 90 days of treatment, increase in N-terminal pro-brain natriuretic peptide has also been found to relate to abnormal LV thickness-to-dimension ratio, which suggests LV remodeling, 4 years later [19]. Other studies have, however, failed to find correlations between early elevation of cTnT and troponin I with cardiac dysfunction at a later stage [20], [21]. Potential alteration in hs-cTnT levels late after treatment in long-term survivors has not been studied previously. To our knowledge, this is the first study to determine using a highly sensitive assay the circulating cTnT levels in adult survivors of childhood leukaemia and to interrogate the clinical determinants and functional implications.

In an adult Caucasian general population, a prevalence of about 37% in men and 13% in women aged younger than 40 years having detectable hs-cTnT has been reported recently [12]. In the present study, the prevalence of detectable hs-cTnT was similar between survivors and controls at about 85–90% in male subjects and 31–35% in female subjects. Importantly, higher levels of hs-cTnT in a population-based cohort were associated with LV structural changes including wall thickening and dilation, LV systolic dysfunction, and subsequent risk for all-cause mortality [12]. In 1,072 middle-aged Japanese men without overt cardiovascular disease, detectable hs-cTnT levels were found in about 81% [13]. The hs-cTnT levels were further shown to be significantly associated with cardiovascular risk factors including age, blood pressure, renal function, current smoking, and LV hypertrophy, and predicted cardiovascular disease risk [13]. A prognostic role of hs-cTnT level is increasingly evidenced in patients with chronic heart failure [22], [23], dilated cardiomyopathy [24], and pulmonary arterial hypertension [25]. Our finding of a positive correlation between LV mass and hs-cTnT level agrees with the previously reported findings [13]. Differences in in LV mass between male and female survivors (70.8±15.2 g/m^2^ vs 61.9±18.0 g/m^2^, p = 0.01) may perhaps also explain the sex differences in hs-cTnT levels, albeit the reported greater susceptibility to anthracycline cardiotoxicity in female patients [4]. This further attests to the need for adopting different cut-off values as discussed below.

Differences in circulating hs-cTnT levels between males and females are well documented [26]. The 99^th^ percentile limit for males has been reported to be about 1.7 times as high as the limit for females [26]. We have therefore derived different cut-off values based on male and female control subjects. A male cutoff value of 7.91 ng/L corresponds to the highest tertile of hs-cTnT levels in the recently reported Japanese study, in which patients tertile cutoffs were respectively ≤2 ng/L, 3 to 4 ng/L, and ≥5 ng/L [13]. This value also corresponds to the 4^th^ highest category of hs-cTnT levels (6.6 to <14 ng/L) in the population-based study that included 3546 adult subjects [12]. These data support our stratification of survivors into two groups based on the non-parametric estimate of 95^th^ percentile in healthy controls.

Clinical variables in survivors associated with increased plasma hs-cTnT levels include cumulative anthracycline dose received and history of leukaemic relapse, stem cell transplant, and cardiac irradiation. Cumulative anthracycline dose and cardiac irradiation are known risk factors for development of late cardiac dysfunction [4], but none of these were found to correlate with indices of cardiac function in the present study (data not shown). This implies perhaps the more sensitive nature of hs-cTnT in the detection of subtle myocardial damage compared with conventional echocardiographic indices or even the newer parameters of myocardial deformation [17]. Our findings further support a risk-stratified long-term screening of cardiac dysfunction according to cumulative anthracycline dosage received and history of cardiac irradiation as advocated by the Cardiovascular Disease Task Force of the Children’s Oncology Group [27].

The association between polymorphism of the *CYBA* rs4673 and higher hs-cTnT levels is intriguing. The *CYBA* gene codes for p22-phox, a subunit that that forms the NAD(P)H oxidase multienzyme complex. The T allele at position 242 leads to a missense mutation His72Tyr. A gene dose-dependent reduction of stimulated NAD(P)H oxidase activity in human neutrophils of T allele carriers has been reported [28]. On the other hand, increase in NAD(P)H oxidase activity in cells transferred with CYPA expression constructs and in neutrophils of human subjects carrying the T allele has also been described [29]. Notwithstanding the conflicting data on the functional consequences of this single nucleotide polymorphism, acute anthracycline-induced cardiotoxicity has been associated with *CYBA* rs4673 polymorphism [5]. Hence, genetic variants in free radical metabolism may play a role not only in predisposing to acute but also chronic anthracycline-induced cardiotoxicity. The observed slightly higher prevalence of *NCF4* GG/AG genotype in survivors than in controls has not been reported previously and needs further clarification. Nonetheless, this genetic variant is not associated with hs-cTnT levels. It is worthwhile noting that genotype distributions of NAD(P)H oxidase and multidrug resistance protein genes in our ethnic Chinese controls differ from those reported in Caucasians [5]. On the other hand, genotypic distributions of *CYBA rs4673*, *RAC2 rs13058338*, *NCF-4 rs1883112*, and *MRP2 rs8187710* in our control subjects are similar to those reported in the Chinese population published in the National Center for Biotechnology Information (NCBI) database (Table 3).

Although factors associated with increased circulating hs-cTnT levels in our survivors have been defined, the mechanisms accounting for the increase are not entirely clear. In long-term survivors with even preserved LV ejection fraction, we have previously shown impairment of LV myocardial deformation [17]. Importantly, elevated hs-cTnT levels were associated with indices of worse systolic and diastolic myocardial deformation. In a mice model of late-onset doxorubicin-induced cardiotoxicity, juvenile exposure to anthracyclines has been shown to impair cardiac progenitor cell function and vascularization and result in greater susceptibility to exercise and ischaemic stress-induced myocardial injury in adult mice [30]. Chronic but reversible injury from stress-induced myocardial strain may hence be a possible culprit leading to transient changes in cell membrane permeability and leakage of cTnT in patients after anthracycline therapy [31]. Anthracycline-induced apoptosis of cardiomyocytes is also recognized [32], although it remains uncertain whether apoptosis leads to troponin release [33]. Taken together, although increased hs-cTnT levels may possibly reflect ongoing cardiomyocyte injury, further studies are required to unveil the mechanisms of cTnT release late after completion of anthracycline therapy.

Several limitations to this study deserve comments. First, this is a case-control study that does not provide data on prognostic implications of elevated hs-cTnT levels. It would be ideal to monitor longitudinally the cardiac and functional status of the survivors through adulthood for determination of the predictive value of hs-cTnT on development of late cardiomyopathy and functional impairment in terms of exercise tolerance. Second, the number of subjects was relatively small and our findings need to be confirmed in larger scale multi-centre studies. This may possibly explain the relatively small, albeit statistically significant, differences between groups I and II patients and the rather weak association between myocardial deformation parameter and hs-cTnT levels. In control subjects, we have nonetheless made efforts to compare the hs-cTnT levels with those reported in the literature [13], [26]. Second, echocardiographic assessment was performed at rest. A recent study has demonstrated reduced LV contractile reserve during exercise stress in children previously treated with anthracycline [34]. Given the reported greater susceptibility to stress-induced myocardial injury in adult mice with juvenile anthracycline exposure [30], it would be worthwhile to determine in long-term survivors the relationship between hs-cTnT levels and LV contractile reserve under stress. Third, we have not assessed myocardial perfusion in our surviving cohort. Myocardial perfusion abnormalities have predominantly been found in patients treated for Hodgkin’s disease and breast cancer [35]. Although none of our survivors have clinical evidence of myocardial ischaemia, subclinical perfusion abnormalities cannot completely be excluded. Nonetheless, acute myocardial ischaemia remains an unlikely explanation for the finding of increased hs-cTnT levels.

In conclusion, increased hs-cTnT levels occur in a significant proportion of adult survivors of childhood leukaemias. Higher levels are associated with larger cumulative anthracycline dose received and history of leukaemic relapse, stem cell transplant, and irradiation involving the heart, genetic variants in free radical metabolism, and worse LV myocardial deformation. Further longitudinal studies are undoubtedly warranted to confirm our preliminary findings, to ascertain the prognostic value of elevated hs-cTnT levels, and to validate the use of hs-cTnT as a biomarker for screening of anthracycline cardiotoxicity in long-term survivors of childhood cancers.
